# Impact Force and Velocities for Kicking Strikes in Combat Sports: A Literature Review

**DOI:** 10.3390/sports12030074

**Published:** 2024-03-06

**Authors:** Daniel Corcoran, Mike Climstein, John Whitting, Luke Del Vecchio

**Affiliations:** 1 Faculty of Health, Southern Cross University, Bilinga, QLD 4225, Australia; 2 Physical Activity, Lifestyle, Ageing and Wellbeing Faculty Research Group, University of Sydney, Camperdown, NSW 2050, Australia; 3 Clinical and Health Services, Faculty of Health, Southern Cross University, Bilinga, QLD 4225, Australia

**Keywords:** kick, martial arts, kinetics, kinematics, measurement, striking, biomechanics

## Abstract

Kicking strikes are fundamental in combat sports such as Taekwondo, karate, kickboxing, Muay Thai, and mixed martial arts. This review aimed to explore the measurement methods, kinematics such as velocities, kinetics such as impact force, determinants, and injury potential of kicking strikes in combat sports. Searches of Academic Search Premier, The Allied and Complementary Medicine Database, CINAHL Plus, MEDLINE, SPORTDiscus, Scopus, and Web of Science databases were conducted for studies that measured kicking velocity and impact force. A total of 88 studies were included in the review. Studies most frequently involved only male participants (49%) aged between 18 and 30 years of age (68%). Studies measuring velocity predominantly implemented camera-based motion capture systems (96%), whereas studies measuring impact force displayed considerable heterogeneity in their measurement methods. Five primary strikes were identified for which foot velocities ranged from 5.2 to 18.3 m/s and mean impact force ranged from 122.6 to 9015 N. Among the techniques analysed, the roundhouse kick exhibited the highest kicking velocity at 18.3 m/s, whilst the side kick produced the highest impact force at 9015 N. Diverse investigation methodologies contributed to a wide value range for kicking velocities and impact forces being reported, making direct comparisons difficult. Kicking strikes can be categorised into throw-style or push-style kicks, which modulate impact through different mechanisms. Kicking velocity and impact force are determined by several factors, including technical proficiency, lower body strength and flexibility, effective mass, and target factors. The impact force generated by kicking strikes is sufficient to cause injury, including fracture. Protective equipment can partially attenuate these forces, although more research is required in this area. Athletes and coaches are advised to carefully consider the properties and potential limitations of measurement devices used to assess impact force.

## 1. Introduction

In striking-based combat sports, competitors commonly use punches, kicks, knee, and elbow strikes [1]. When utilising striking techniques, a competitor must deliver strikes quickly enough to bypass an opponent’s defence or protective reaction [2,3] and impact with enough force to weaken or incapacitate an opponent [4]. Strikes that meet these criteria also influence judging decisions [4], increasing the likelihood of victory by decision.

Combat sports athletes have traditionally been profiled based on anthropometry and physiological capacities such as limb strength and cardiovascular fitness [5,6,7]. However, the research on quantification and performance profiling of sport-specific qualities, including striking velocity and impact force, remains comparatively unexplored [8]. While only upper limb strikes are permitted in some combat sports like boxing, disciplines such as Taekwondo (TKD), karate (KA), kickboxing (KB), Muay Thai (MT), and mixed martial arts (MMAs) allow the use of both upper and lower limb strikes. In disciplines where a combination of upper and lower limb strikes is permitted, the frequency at which these strikes are used varies. In TKD, kicking strikes are the dominant method of striking [9], whereas in KA, punches occur at a higher frequency than kicking strikes [10]. Nonetheless, delivering high-velocity kicks is a crucial tactical factor and an essential determinant of competition success [11,12,13,14,15]. Across disciplines, a range of kicks are employed, including roundhouse, front, back, side, and axe kicks [16,17,18]. Specifically in TKD, the roundhouse kick is the most commonly used [19] and has been reported to be the most frequent cause of concussion during competition [20]. Furthermore, kicks to the head are considered tactically important in combat sports, as disrupting an opponent’s ability to compete through knockout is the most direct route to victory [16].

Various investigations have explored aspects of kicking performance, including the kinematic stages of kicking [21,22,23,24,25], interdisciplinary performance comparisons [24,26,27], the effect of training interventions [28,29,30], and physical determinants of kicking performance [31,32]. Furthermore, numerous studies have explored injury and injury prevalence in combat sports involving kicking [33,34,35]. Nonetheless, despite the growing popularity of combat sports, the scope of the scientific literature has not kept pace with this trend [36]. To date, no literature review has investigated the velocity and force characteristics across the range of kicking strikes used in combat sports or explored the potential injury these forces may cause. Considering the tactical importance of delivering high-velocity, high-force strikes, quantifying the variety of kicks performed in combat sports can enhance the understanding of kicking performance across combat sports and assist in improving safety practices, training interventions, talent identification, and elite performance [37,38]. Therefore, this review aimed to summarise the velocities and impact forces reported in studies on kicking strikes in combat sports. It additionally aimed to explore their measurement methods, kinematics, kinetics and potential performance determinants and evaluate the theoretical injury risk associated with such strikes.

## 2. Materials and Methods

### 2.1. Literature Search Strategy

A comprehensive electronic database search focused on kicking strikes was conducted on 30 June 2023. The search encompassed seven databases: Academic Search Premier, The Allied and Complementary Medicine Database, CINAHL Plus, MEDLINE, SPORTDiscus, Scopus, and Web of Science. The following combination of search terms was used: “martial arts” OR “combat sports” OR “combat sport” OR karate OR kickboxing OR kick-boxing OR “kick boxing” OR “mixed martial arts” OR mma OR taekwondo OR TKD OR “Tae-kwon-do” AND force OR velocity OR impact AND kick. Truncated versions of the search term ‘kick’ were used to ensure all relevant studies were identified. Search results from each database were combined, and duplicates were removed. A further search was performed in the reference lists of included papers for additional relevant articles.

### 2.2. Eligibility Criteria

The inclusion criteria for articles were (a) reported linear foot velocities or impact force from kicking strikes delivered by human participants, (b) articles available in the English language, and (c) articles published in a peer-reviewed journal. The exclusion criteria included articles that did not involve human participants, reported kinetic or kinematic qualities other than linear foot velocity or impact kinetics, were in languages other than English, or were secondary research. Additionally, studies using measurement methods that reported velocity and impact force in units other than metres per second (m/s) for velocity and Newtons (N) for force were also excluded.

### 2.3. Data Extraction

After identification of eligible studies, data including participant characteristics, measurement method, strike measured, and reported value (foot velocity or impact force) were extracted into tabular form (See Supplementary Material Tables S3 and S4). When data were only reported in graphs, values were extracted using WebPlotDigitizer (Version 4.7) [39].

### 2.4. Literature Quality Assessment

A critical appraisal of all articles included in this review was carried out using the Appraisal tool for Cross-Sectional Studies (AXIS). The AXIS tool, specifically designed for cross-sectional studies, consists of 20 domains to determine the quality of individual investigations [40]. The AXIS tool was modified for this review by removing questions 7, 13, and 14, as non-response was deemed inappropriate for the studies included [17]. Following the assessment of each investigation, AXIS scores were converted to percentages to reflect the quality of the study [41]. Three scoring brackets for studies were assigned for the studies as follows: Good quality (≥74%), Fair quality (≥55 to ≤73.9%), and Poor quality (≤54.9%) [41]. 

## 3. Results

**Figure 1:**
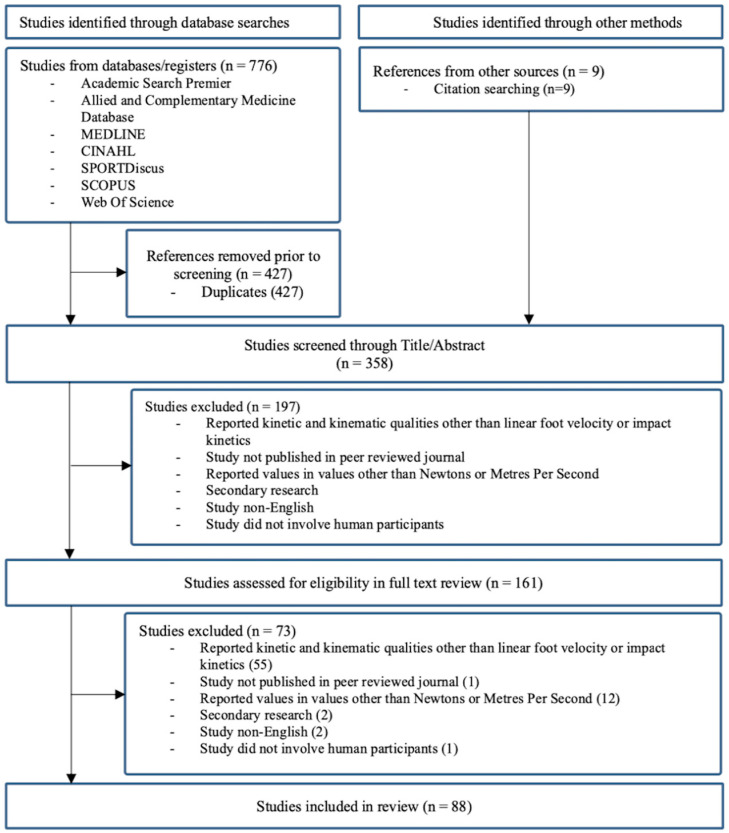
Flow chart of the article retrieval process.

The literature search across the seven databases retrieved a total of 776 results. Following the removal of duplicates, subsequent title and abstract screening, and full-text screening, 79 articles were deemed suitable for inclusion. An additional nine relevant and eligible texts were identified within the reference sections of the included articles. In total, 88 studies met the inclusion criteria and were included in the final synthesis. Figure 1 details the process of article retrieval and inclusion.

### 3.1. Literature Quality

All studies were evaluated and scored according to the 17 criteria of the modified AXIS assessment. Of the evaluated studies, the majority (*n* = 68, 77.3%) were rated as ‘Good quality’, 16 (18.2%) as ‘Fair’ quality, and 4 (5.0%) as ‘Poor’ quality. Two studies returned an AXIS score of 100% [42,43]. Justification of sample size was infrequent among the included studies, with only 11 studies providing rationale for the sample size used [12,21,25,26,31,42,43,44,45,46,47]. Likewise, measures of internal consistency were few, with only 14 studies reporting any measure of consistency in their results [11,28,31,43,44,48,49,50,51,52,53,54,55,56]. The modified AXIS assessment for individual studies is available in the Supplementary Material (Table S2).

### 3.2. Study Participants and Characteristics

Of the 88 studies identified, participant sample sizes ranged from 1 to 49 participants. The mean, median, and mode for sample sizes across all studies was 15.5, 14, and 6, respectively. One investigation did not report a sample size [57]. Approximately half of the studies identified recruited only male participants (*n* = 43, 49%). Fewer studies recruited male and female participants (*n* = 25, 28%), and a very small proportion recruited only female participants (*n* = 2, 2%). The remainder of investigations failed to report the specific sex breakdown of their participant group (*n* = 18, 20%). Regarding mean participant age, four categories were identified: <18, 18–30, 30–50, and >50 years of age. The majority of the studies (*n* = 60, 68%) used participants aged 18 to 30. Studies involving participants <18 and 30–50 were less common (*n* = 6, 7%, and *n* = 4, 5%, respectively). A small number (*n* = 8, 9%) of studies involved participants from multiple age brackets, and only one study involved participants >50 years of age [58]. Three categories of mean participant training experience were identified: <4, 4–10, and >10 years of training experience. The 4–10 and >10 years categories were the most frequently reported (*n* = 24, 27%, and *n* = 20, 23%, respectively). Studies involving participants of <4 years or experience were fewer (*n* = 7, 8%), and studies involving multiple categories were slightly less still (*n* = 6, 7%). Studies that did not report experience in years were more prevalent (*n* = 31, 35%). Detailed study characteristics, including sample size, participant sex, age, and training experience, are available in the Supplementary Material (Tables S3 and S4).

### 3.3. Measurement of Kicking Velocity and Impact kinetics

Among the studies included in the review, 56 reported velocity values, 21 reported impact force values, and 11 reported both velocity and impact force. The measurement of velocity was predominantly performed using camera-based motion capture systems (*n* = 64, 96%), followed by ground sensor and racket systems (*n* = 2, 3%) and dual-beam laser systems (1%, *n* = 1). The measurement of impact forces was most frequently performed using various force sensors (*n* = 14, 44%), followed by force plates (*n* = 7, 22%), accelerometers (*n* = 5, 16%), strain gauges (*n* = 3, 9%), calculation from motion capture (*n* = 2, 6%), and spring balance (*n* = 1, 3%). The reliability of velocity and impact force measurement, as indicated by the intraclass correlation coefficient (ICC) and Cronbach’s alpha, was reported in 13 studies [11,28,43,44,48,49,50,51,52,53,54,55,56].

### 3.4. Kicking Velocity and Impact Force

**Figure 2:**
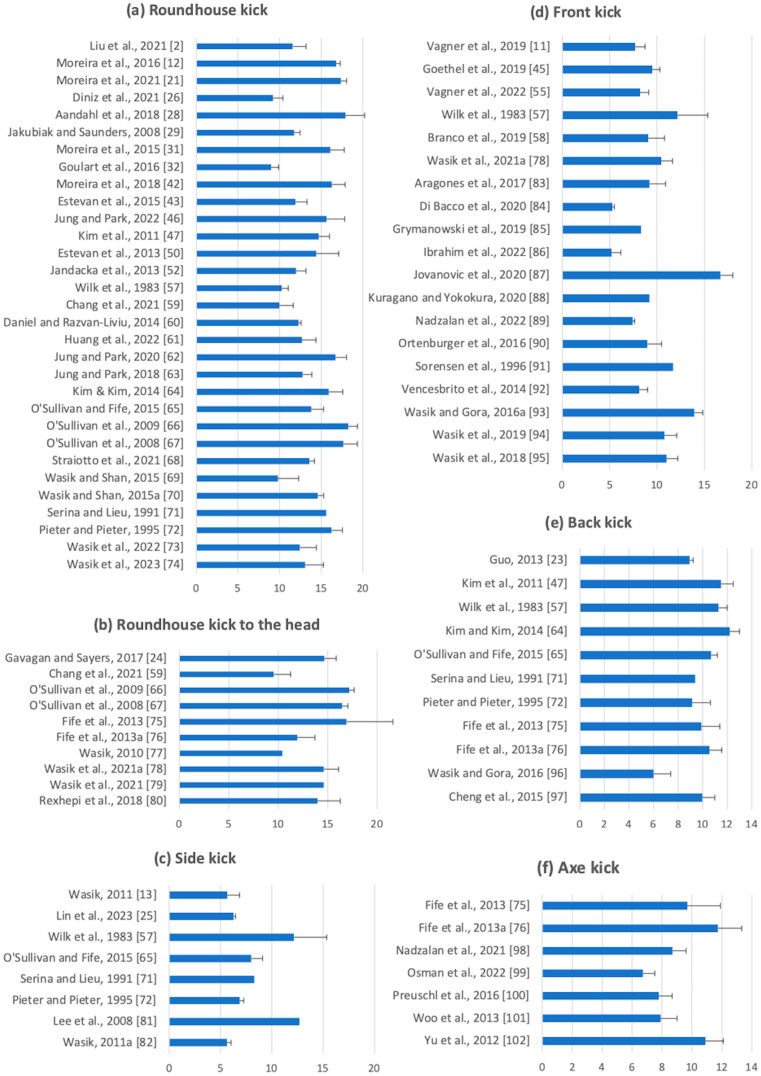
Means and standard deviations of peak linear foot velocity (m/s) for kicking strikes in combat sports: (**a**) roundhouse kick, (**b**) roundhouse kick to the head; (**c**) side kick, (**d**) front kick, (**e**) back kick, (**f**) axe kick. Where an error bar is absent, only a maximum velocity value was reported [2,11,12,13,21,23,24,25,26,28,29,31,32,42,43,45,46,47,50,52,55,57,58,59,60,61,62,63,64,65,66,67,68,69,70,71,72,73,74,75,76,77,78,79,80,81,82,83,84,85,86,87,88,89,90,91,92,93,94,95,96,97,98,99,100,101,102].

**Figure 3:**
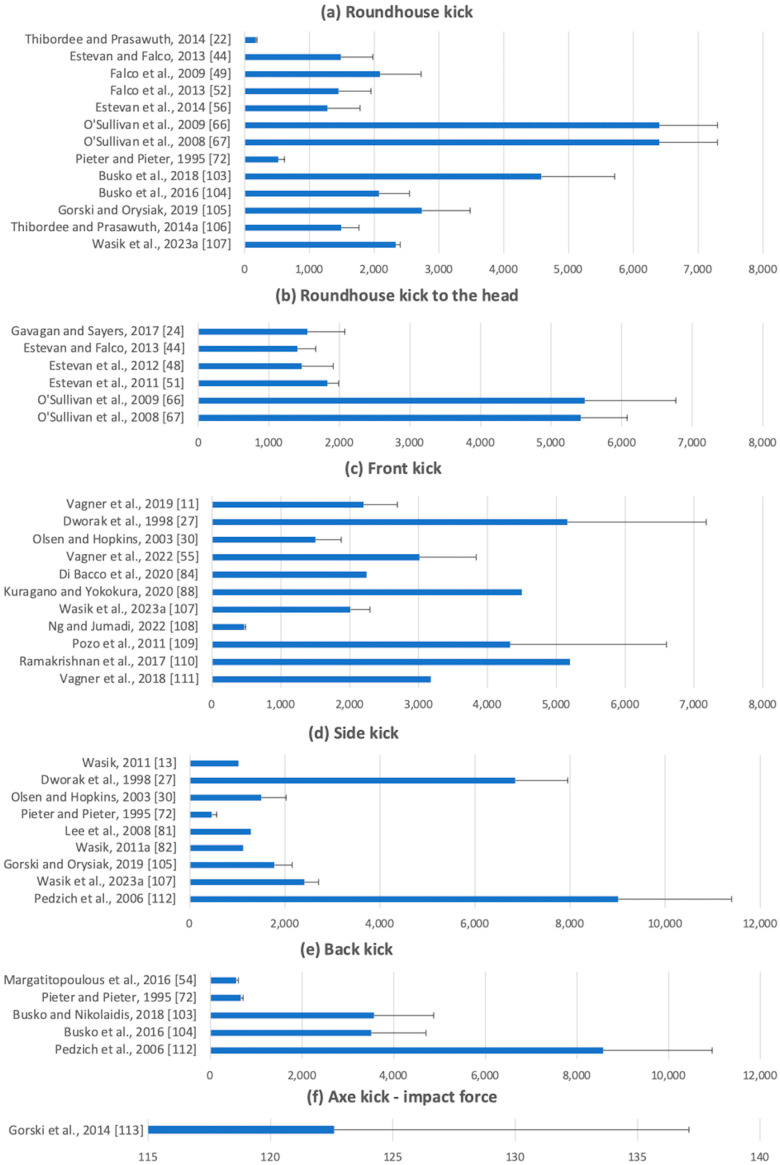
Means and standard deviations of impact force (N) for kicking strikes in combat sports: (**a**) roundhouse kick; (**b**) roundhouse kick to the head; (**c**) front kick; (**d**) side kick, (**e**) back kick; (**f**) axe kick. Where an error bar is absent, only a maximum impact force value was reported. Where only N/KG was reported, an average participant weight of 75 kg [121] was used to calculate impact force [11,13,22,24,27,30,44,48,49,51,52,54,55,56,66,67,72,81,82,84,88,103,104,105,106,107,108,109,110,111,112].

Velocity and impact force values from the literature search are presented in Figure 2 and Figure 3. The velocity of kicks was reported in 31 instances for the roundhouse kick [2,12,21,26,28,29,31,32,42,43,44,46,47,53,57,59,60,61,62,63,64,65,66,67,68,69,70,71,72,73,74], 10 for the roundhouse kick to the head [24,59,66,67,75,76,77,78,79,80], 8 for the side kick [13,25,28,57,65,71,72,81,82], 19 for the front kick [11,45,55,57,58,78,83,84,85,86,87,88,89,90,91,92,93,94,95], 11 for the back kick [23,47,57,64,65,71,72,75,76,96,97], and 7 for the axe kick [75,76,98,99,100,101,102]. Impact force was reported in 13 instances for the roundhouse kick [22,44,49,52,56,66,67,72,103,104,105,106,107], 6 for the roundhouse kick to the head [24,44,48,51,66,67], 11 for the front kick [11,27,30,55,84,88,107,108,109,110,111], 9 for the side kick [13,27,30,72,81,82,105,107,112], 5 for the back kick [54,72,103,104,112], and 1 for the axe kick [113]. Participants from eight combat sports disciplines were reported. TKD was the most frequently reported discipline (*n* = 69), followed by KA (*n* = 12), MT (*n* = 3) and KB (*n* = 2). Krav Maga, Nihon-Kempo, Silat, and Yongmudo were each reported singularly. Interdisciplinary comparison was conducted in four studies [24,26,27,66], and five studies did not specify the training disciplines of participants. Detailed characteristics of these studies, including participant sample, measurement method, type of strikes measured, and reported values, are available in the Supplementary Material (Tables S3 and S4).

Across all disciplines, kicking variations, and techniques, foot velocities ranged from 5.2 to 18.3 m/s. Amongst papers reporting impact force, the values reported ranged from 122.6 N to 9015 N. Kicks, including those of similar styles, demonstrated a range of foot velocities. Roundhouse kicks produced velocities ranging from 6.9 to 18.3 m/s; side kicks produced velocities ranging from 5.6 to 12.7 m/s; back kicks produced velocities ranging from 6.0 to 12.2 m/s; front kicks produced velocities ranging from 5.2 to 16.7 m/s; and axe kicks produced velocities ranging from 6.5 to 10.9 m/s. In terms of impact force, roundhouse kicks’ impact force ranged from 172.0 to 6400 N; side kicks ranged from 461.8 to 9015 N; back kicks ranged from 562.4 to 8569 N; front kicks ranged from 466.6 to 7790 N; and axe kicks were reported at 122.6 N. Of the studies included in this review, the kick with the highest foot velocity was the roundhouse kick (18.3 m/s), and the kick with the highest impact force was the side kick (9015 N).

## 4. Discussion

This review identified the velocities and impact force of common combat sports kicking strikes. Five commonly measured kicking strikes across eight disciplines were identified: the roundhouse kick, front kick, side kick, back kick, and axe kick. Additionally, various kicks unique to individual combat sports were identified, including the ‘Tornado’ kick [65], the ‘Thrashing’ kick [47], several spinning kicks [71,76], and variations of the axe kick [75,76,101]. Of all the kicks identified in this review, the side kick generated the highest impact force (9015 N) [112], and the roundhouse kick generated the highest peak foot velocity (18.3 m/s) [66]. Participants in the studies identified were predominantly male, with 49% of studies reporting only male participants. Studies with only female participants studies were rare (2%). Participants were most frequently aged between 18 and 30 (68%), with the 4–10 and >10 years of training experience categories encompassing 50% of all participants. TKD was the most frequently studied discipline, followed by KA, MT, and KB. Krav Maga, Nihon-Kempo, Silat, and Yongmudo were reported in single instances. When compared with upper limb strikes, the side kick produced a force 158% greater than that of the reported highest force upper limb strike, the straight punch (5358 N) [114]. Similarly, the roundhouse kick, the most common kick identified, generated 119% of straight punch force at 137% of its velocity (18.3 m/s compared to 13.4 m/s) [115]. Considering that the lower limb mass in combat sports practitioners is often more than 2.5 times greater than that of upper limb mass [116], it may be surprising that the velocities of some kicking strikes were higher than those of common upper limb strikes. This effect is likely attributable to the summative involvement of the large lower limb segments, including the pelvis, hip, and thigh [24]. Furthermore, the mass of these segments may also explain the larger magnitudes of impact force seen in kicking strikes [66]. When interdisciplinary studies were conducted, KB displayed higher impact force than KA [27] and KA demonstrated significantly higher kicking velocity than TKD and MT [26]. However, other comparative studies found no significant differences in velocity and impact force between TKD, KA, and MT [24], or between TKD and Yongmudo [66]. The limited number of comparative studies and varied results within these studies restricted the ability to draw definitive conclusions regarding interdisciplinary differences.

### 4.1. The Measurement of Kicking Velocity and Impact Force

The measurement of velocity in the reviewed studies was primarily conducted using camera-based motion capture systems, considered the ‘gold standard’ for kinematic analysis [117]. Conversely, impact force measurement showed considerable variety, with studies using accelerometers, force plates, force sensors, motion capture, a spring balance, and strain gauges. This variation in measurement devices may explain the disparities in the force values reported across different investigations. Previous studies exploring upper limb striking have suggested that diverse results in striking force may be explained by differences in padding applied to striking surfaces [118] or by the increased rigidity of a device (i.e., force plates) inflating impact force values [119]. A previous review into combat sports striking proposed that devices used to measure impact force should be categorised based on the following properties: those that measure force either directly (i.e., force plate) or indirectly (i.e., accelerometer) and those that are inertially relevant or irrelevant, meaning they mimic or do not mimic the movement of human structure on impact [8]. Among the studies that measured force (*n* = 32), only 13 used inertially-relevant devices during testing [44,48,49,51,52,56,65,66,72,103,104,105,113].

Regarding the reliability of the measurement methods, while both direct and indirect methods have demonstrated good reliability in measuring striking impact forces [8], only 15% (*n* = 13) of studies identified in this review reported any measure of reliability for their impact force or velocity measurements, raising concerns about the consistency and repeatability of the reported findings. ICC was the most commonly reported statistic for assessing measurement reliability. The reported ICC values ranged from 0.53 to 0.97 for foot velocity [11,28,43,50,53,55] and from 0.61 to 0.98 for impact force [11,44,48,51,52,54,55]. These ranges indicate moderate to excellent reliability when reported [120]. The variability in ICC values, coupled with the limited number of studies reporting them, presents a substantial barrier regarding the standardisation of measurements within the field. This inconsistency hinders the comparison and in-depth analysis between studies [66,105,112], underscoring the requirement for more uniform measurement methodologies.

Numerous pitfalls in striking measurement technologies have been identified, suggesting that these technologies require specific methodological requirements to produce accurate data and overcome inherent limitations. Researchers and practitioners using these technologies must be aware of and adept at addressing various issues. These include aberrant movements of kinematic markers [53,100], differences in protective layers applied on devices that dampen impact force [111,112], and variabilities or errors in sensor positioning [68]. These nuances, coupled with the absence of a universally recognised ‘gold standard’ methodology for measuring striking performance, especially regarding impact force [4,8], pose a significant challenge in standardising the assessment of striking performance. 

### 4.2. Kinematics and Impact Kinetics of Kicking Strikes

Kicking strikes in combat sports possess distinct kinematic patterns that influence their velocity and impact characteristics. The individual kinematic patterns for each kicking strike explored in this review are detailed in Table 1. Kicking strikes can be categorised into two kinematic groups: throw-style kicks (such as the roundhouse kick) or push-style kicks (such as the front, side, and back kick) [47]. These two categories are also known as ‘swing’- or ‘thrust’-style kicks [71]. Throw-style kicks involve a sequence of hip flexion followed by rapid knee extension [47], striking the target with the foot instep [71]. A vital aspect of the throw-style kick is the proximal-to-distal movement pattern, in which movement begins at the hip and progresses sequentially distal, with each segment attaining higher velocity than the previous segment [26,43,62,74]. This sequential motion results in a ‘whip’- or ‘flail’-like action [69,91], transferring forces down the lower limb, resulting in high impacts on collision. Conversely, push-style kicks are characterised by a more synchronous motion, in which both the hip and the knee extend almost simultaneously on impact [47], striking an opponent with the plantar surface of the heel [71]. Axe kicks and other discipline-specific strikes, such as the thrashing kick, appear to incorporate elements of both categories, potentially in an attempt to overcome the lower velocity typically associated with push-style kicks [47,102]. 

Kinetically, throw- and push-style kicks are suggested to generate impact forces through different mechanisms [105]. Throw-style kicks are proposed to rely on the proximal-to-distal movement pattern [105], with one study reporting a moderate to good correlation between foot segment velocity and impact force [24]. Subsequently, higher strike velocity would likely result in higher impact force. However, this correlation varied across studies, with one investigation finding an insignificant correlation between strike velocity and impact in males but a significant correlation in females [72]. As throw-style kicks are complex movements requiring coordination between multiple body segments, they likely rely on practitioner skill to utilise proximal-to-distal sequencing effectively to modulate impact forces [51]. In contrast, push kicks appear to modulate impact forces by increasing the body mass involved in the strike, with multiple investigations correlating body mass with impact force [11,110,112]. Since involving more mass in strikes is simpler than enhancing technical skill, push-style kicks have demonstrated improved impact force in untrained participants following a single instructional session [84]. A similar pattern of increasing body mass involved in a strike to improve impact force has been suggested in throw-style kicks. Falco et al. [49] found a significant correlation between body mass and impact force in the roundhouse kick. However, this correlation was only identified in novices, leading the authors to conclude that less skilled practitioners may increase body mass involved in a strike to compensate for technical deficiency.

### 4.3. Determinants of Kicking Velocity and Impact Force

The determinants of kicking velocity and impact force can be categorised into four domains based on the studies identified in this review: technical proficiency, lower body strength and flexibility, effective mass, and target factors. Studies exploring the effect of technical efficiency have noted that those of higher skill qualification can generate higher foot velocity [12,21,45,68,92,102] and impact force [49,55,110] than those of lower skill. 

Suggested mechanisms include superior utilisation of proximal-to-distal motion [49], effective use of body mass [110], higher muscular activation [42,92], and enhanced coordination [45,58]. Lower body strength and flexibility also affect kicking performance, with hip muscular strength [11,31,103], jumping performance [31,32,54], and flexibility [102,108] all being identified as factors influencing kicking performance. Lower body strength likely exerts its effect by increasing an athlete’s ability to create ground reaction forces, potentiating final foot velocity and impact force [12,60], whereas flexibility potentiates length–tension relationships of musculature, increasing kicking effectiveness [102]. However, as athletes fatigue, both the speed [87] and kinematic quality of the kick reduces [83], indicating that athlete endurance also plays a role in kicking performance.

When discussing mass in the context of striking kinetics, it is essential to differentiate between body mass and the effective mass of a strike. As entire body mass cannot be involved in a strike, the portion that actively contributes is termed the ‘effective mass’ [107]. Multiple studies have demonstrated a correlation between kicking impact force and body mass [72,112]. While the correlation between total body mass and effective mass has not been reported in kicking strikes, and a study on punching strikes found only a slight association between the two factors [115]. Only one study identified in this review specifically explored effective mass and its relationship to kicking strikes, reporting on the magnitudes to which effective mass contributed to front, side, and roundhouse kicks [107]. The amount of effective mass involved in a strike is suggested to depend on an athlete’s skill level, with skilled athletes able to time stiffening of the striking limb to occur on or immediately before impact, thereby increasing effective mass and subsequent impact force [97,107]. The neuromuscular action believed to create this stiffening has been referred to as a “contraction-relaxation-contraction strategy” [92]. This strategy involves an initial contraction to start the kicking motion, a relaxation as the limb accelerates, and a final contraction to create limb stiffness on impact [92]. Studies on this stiffening effect in kicking are limited. However, research investigating the roundhouse kick found increased ankle joint rigidity and heightened hamstring activation on impact when comparing high- and low-impact kicks [106]. The authors proposed that increased hamstring activation could serve to stiffen the knee joint and enhance impact force [106]. This stiffening function is important in athlete testing contexts, as without a target necessitating this stiffening effect on impact, athletes tend to produce higher kicking velocities [70,90,93,94,95], raising concerns about the ecological validity of these measurements [79].

When evaluating target-related factors, both the distance from and size of the target appear to affect kicking impact and velocity. A reduction in target size is associated with a decrease in kicking velocity, a modification likely made to accommodate increased accuracy demands [78,79,93]. This phenomenon, known as the ‘speed-accuracy trade-off’, has been reported in multiple studies [78,93,94]. This effect may explain why kicks aimed at the head tend to produce lower impact force and velocity than those targeting the trunk [66,67], likely due to the need for more precise targeting, which necessitates a reduction in absolute velocity. In assessing distance from a target, numerous studies have shown that adjusting stance angle, thereby changing the distance of the striking limb from the target, can influence velocity and impact force. Several studies demonstrated this effect, showing that body angulation of 45–90 degrees relative to the target yielded higher kicking velocities than square-to-target orientations [2,46,50,53,62]. These findings were noted to be minimal in some instances [2,53]. The influence of stance angle is likely attributable to the increased summation of body segment rotations that contribute to the kicking action, an effect that is less potent at lower angles of attack [46]. Additionally, in scenarios where practitioners could choose their preferred striking distance, they achieved higher velocities than when the distance was predetermined [62]. Concerning impact force, it has been suggested that an increased distance from a target allows more time for the striking limb to accelerate before impact, potentially increasing impact force [105]. However, the interplay between distance and impact appears to be more complex. While certain studies have shown that individuals of a higher skill level can maintain impact force across multiple distances [44,49], other research has reported different findings relating to kicking distance and skill level. One study observed lower kicking impact forces at a longer distance for higher-skill athletes only [52], while another found no significant difference in impact force between skill levels at an intermediate kicking distance [51]. Interestingly, a study analysing impact force across weight classes revealed that heavyweight and lightweight athletes produced lower impact force from longer distances, while welterweight athletes maintained similar force across multiple distances [48]. This finding suggests that body mass may interact with distance to influence impact force. Although varying methodologies between these studies may account for the contrasting results, these findings indicate that while skill level may diminish the effect of distance on impact force to a certain extent, the absolute effect remains unclear.

### 4.4. Injury Potential from Kicking Strikes

Injury is a common occurrence in combat sports, with some disciplines reporting that 28.6 per 100 bouts result in injury to a competitor [122]. Common injuries include contusions, lacerations, soft tissue injuries, concussions, fractures, and dislocations [35]. As the head is a tactically important target in striking sports, facial fractures have been reported to account for a large portion of head and neck injuries in sports involving kicks to the head [35]. Considering the force tolerances of the facial bones [123], the highest reported impact force from a roundhouse kick to the head could potentially fracture the nasal bone, maxilla, mandible, zygoma, and the lateral and temporoparietal regions of the skull. As competitors adjust their stance to defend their facial structures with their arms during an opponent’s attack, this can lead to upper limb fractures [124]. For example, the impact of a roundhouse kick well exceeds the reported tolerance range of the forearm bones (670–3550 N [125]). If a competitor can bypass an opponent’s upper limb defences, the ribs become a common impact area, resulting in the third most common fracture site behind the head and upper limbs in certain disciplines [33]. Studies on motor vehicle accidents report that rib fractures can result from forces between 1200 and 5900 N [126], indicating that a well-placed roundhouse, side, or back kick can generate impact forces equivalent to those in a severe motor vehicle accident. Fractures of the lower extremity occur at a much lower prevalence than those of the upper extremity and head [127]; however, kicks targeting the leg would need to generate impact at forces between 4110 and 8450 N and 5000 and 8230 N to fracture the tibia or femur, respectively, as indicated by cadaver studies [128].

Given the potential for injury from striking impacts, protective equipment has been made mandatory in some disciplines involving kicking strikes [129,130]. Despite this, injury prevalence research has indicated that only a limited proportion of participants (26.15%) reported using protective equipment during training sessions in which injury occurred [34]. The use of protective equipment such as hand protection and headgear likely aids in preventing common injuries such as lacerations and fractures, although their effect on concussions is less conclusive [131]. Research into the force-attenuating effects of protective equipment has demonstrated that implementing gloves and head protection can reduce impact forces by up to 67% during simulated head strikes [132]. However, beyond head protection, few studies have examined the force-attenuating effects of other protective equipment used in combat sports, such as body, forearm, foot, or shin protection. One study exploring this effect demonstrated that a body protector reduced impact force by 31.9–75.3% during simulated kicking strikes [133]. This study also found that the attenuation effect decreased as impact forces increased, supporting the suggestion that protective equipment may require enhanced absorption capabilities relative to the expected impact magnitude to improve athlete safety [134]. A similar study investigated the force-attenuating effect of forearm, shin, hand, and foot protection during simulated strikes [135]. The study found varying levels of force attenuation among different brands of protective equipment and reported that under high-impact conditions, the equipment did not reduce impact force below theoretical thresholds for fracture or soft tissue injury. The authors further suggested that improvements in protective equipment are necessary to reduce impact force to <2000 N to prevent severe injuries from striking impact [135]. These findings suggest that kicking strikes generate impact forces sufficient to fracture various bony structures and protective equipment appears to offer a degree of force attenuation during impact. However, further research and development of this equipment is essential to improve the safety of participants involved in combat sports and to reduce injury prevalence.

### 4.5. Strengths and Limitations

This review paper demonstrates several strengths in examining the velocities and impact forces of kicking strikes in combat sports. It presents a comprehensive summary of these values, encompassing a wide range of studies, and offers a thorough overview of the existing literature. This review offers readers a holistic understanding of the velocity and impact outcomes associated with kicking strikes in combat sports. Additionally, it provides valuable insight into the participant cohorts studied and the varied methodologies employed to measure velocity and impact force values within these cohorts. By summarising the diverse measurement approaches found in the literature, this review offers valuable insight into the range of methods used in combat sports research. This review enables researchers and practitioners to understand the various measurement methodologies and the kinematic and impact determinants involved in kicking strikes.

While this review summarises the velocity and impact force of kicking strikes in combat sports, caution is advised in comparing or analysing across studies, as the heterogeneity of methodologies often prohibits direct comparison [66,105,112]. Additionally, few studies included in this review reported justification for their sample size (which was often small) or the consistency of measures in their assessment methodologies, raising concerns about the internal validity of these investigations. The external validity of the values reported within this paper are also contentious, as males and females were analysed jointly in this review. Furthermore, as participant testing was primarily conducted in a laboratory environment, translation into live competition may be limited. This issue of ecological validity has been previously noted, as no laboratory test can fully replicate a real competition scenario [2,11,46,62,63,85,106]. Careful consideration should be taken when applying these findings to general populations, as participants were most frequently male, aged 18–30, with 4–10 years of training experience. Additionally, some studies reviewed involved elite practitioners whose performance outputs may exceed that of less skilled participants [59,61]. The potential for injury from kicking strikes is theoretical in nature, and injury, including fracture, depends on more than force alone and is also contingent on other factors including strike vector direction, velocity, and contact time [136]. Finally, the authors acknowledge that excluding non-peer-reviewed publications, which may potentially include eligible studies, was a necessary criterion to maintain the quality standards of this review process.

## 5. Conclusions

Kicking strikes in combat sports are commonly evaluated by measuring foot velocity and impact force. Kicks commonly measured included the roundhouse, front, side, back, and axe kick. The roundhouse kick was the most frequently measured, likely due to its high velocity and high-impact force. Combat sports kicks can generally be categorised into two types: throw-style kicks, which likely modulate impact force through effective use of the proximal-to-distal movement pattern, and push-style kicks, which modulate force through increased use of body mass. The determinants of kicking velocity and impact force include technical proficiency, lower body strength and flexibility, effective mass, and target factors. The impact force from kicking strikes has the potential to cause injury, including the fracture of several bony structures. Protective equipment can attenuate impact force during kicking strikes and should be considered for athlete safety. However, further research is needed to ensure that this equipment can sufficiently attenuate the forces from high-impact kicks to prevent injury.

While this review has provided valuable insights, the heterogeneity of measurement methodologies presents a major limitation within the literature, particularly the measurement of impact force. This diversity is demonstrated by the wide variation in reported values of kicking velocities and impact forces among different kicking strikes, posing considerable challenges for direct comparison. Further research is needed to validate and standardise striking measurement methodologies to deepen the understanding of factors that contribute to high-velocity and high-force kicking strikes.

## Figures and Tables

**Table 1:** Kinematic patterns of kicking strikes.

Roundhouse kick	Initiates with forward movement towards an opponent with pelvic rotation, followed by hip abduction, hip flexion, and knee flexion, finishing with knee extension of the striking leg to strike the opponent with the instep of the foot [24,77].
Front kick	Initiates with hip and knee flexion to a near parallel position, followed by rapid extension of the hip and knee horizontally toward the target, striking with the foot [11,89,91,109].
Side kick	Initiates with striking leg lifting off the ground and pelvic tilt away from the target, accompanied by simultaneous hip flexion, abduction, and knee flexion, followed by hip and knee extension to strike the target with the heel [13,25,81,82].
Back kick	Initiates with body rotation away from the target, combined with simultaneous hip flexion, abduction, and knee flexion, finishing with hip and knee extension to strike the target with the heel [64,96].
Axe kick	Initiates with lifting the striking leg into flexion with simultaneous knee extension as the leg ascends, followed by a rapid hip extension to strike the target with the heel [98,99,102].

## Supplementary Materials

The following supporting information can be downloaded at: https://www.mdpi.com/article/10.3390/sports12030074/s1. Table S1: Search strategy, Table S2: Appraisal tool for Cross-sectional Studies (AXIS) assessment for included studies, Table S3: Summary of studies that reported kicking strike velocity, Table S4: Summary of studies that reported kicking strike impact force.
