# Supplementary material for: Impact Force and Velocities for Kicking Strikes in Combat Sports: A Literature Review
Source: Sports (Basel). 2024 Mar 6;12(3):74. doi: 10.3390/sports12030074 (PMC10974023; doi:10.3390/sports12030074)
Supplement: Supplementary file 1 [file sports-12-00074-s001.zip › sports-2859797-supplementary.pdf]

Table S1. Search strategy

The below search terms were used in the following electronic databases: Academic Search Premier, The Allied and Complementary Medicine Database, CINAHL Plus, MEDLINE, SPORTDiscus, Scopus and Web Of Science. Search was limited to articles in English language and published in peer reviewed publication.

Database	Search strategy
SCOPUS	(TITLE-ABS-KEY ("martial arts" OR "combat sports" OR "combat sport" OR karate OR kickboxing OR kick-boxing OR "kick boxing" OR "mixed martial arts" OR mma OR taekwondo OR tkd OR "Tae-kwon-do") AND TITLE-ABS-KEY (force OR velocity OR impact) AND TITLE-ABS-KEY (kick*)) AND (LIMIT-TO (DOCTYPE , "ar")) AND (LIMIT-TO (LANGUAGE , "English"))
Web Of Science - all databases	"martial arts" OR "combat sports" OR "combat sport" OR karate OR kickboxing OR kick-boxing OR "kick boxing" OR "mixed martial arts" OR mma OR taekwondo OR TKD OR "Tae-kwon-do" (Topic) and force OR velocity OR impact (Topic) and kick* (Topic) and Preprint Citation Index (Exclude – Database) and Article (Document Types) and English (Languages)
CINAHL Plus	("martial arts" OR "combat sports" OR "combat sport" OR karate OR kickboxing OR kick-boxing OR "kick boxing" OR "mixed martial arts" OR mma OR taekwondo OR TKD OR "Tae-kwon-do") AND (force OR velocity OR power OR impact) AND kick*<br><br>Limiters: English language, Peer reviewed
AMED - The Allied and Complementary Medicine Database	("martial arts" OR "combat sports" OR "combat sport" OR karate OR kickboxing OR kick-boxing OR "kick boxing" OR "mixed martial arts" OR mma OR taekwondo OR TKD OR "Tae-kwon-do") AND (force OR velocity OR power OR impact) AND kick*<br><br>Limiters: English language, Peer reviewed
Academic Search Premier	("martial arts" OR "combat sports" OR "combat sport" OR karate OR kickboxing OR kick-boxing OR "kick boxing" OR "mixed martial arts" OR mma OR taekwondo OR TKD OR "Tae-kwon-do") AND (force OR velocity OR power OR impact) AND kick*<br><br>Limiters: English language, Peer reviewed
MEDLINE	("martial arts" OR "combat sports" OR "combat sport" OR karate OR kickboxing OR kick-boxing OR "kick boxing" OR "mixed martial arts" OR mma OR taekwondo OR TKD OR "Tae-kwon-do") AND (force OR velocity OR power OR impact) AND kick*<br><br>Limiters: English language, Peer reviewed
SPORTDiscus	("martial arts" OR "combat sports" OR "combat sport" OR karate OR kickboxing OR kick-boxing OR "kick boxing" OR "mixed martial arts" OR mma OR taekwondo OR TKD OR "Tae-kwon-do") AND (force OR velocity OR power OR impact) AND kick*<br><br>Limiters: English language, Peer reviewed

Table S2. Appraisal tool for Cross-sectional Studies (AXIS) assessment for included studies

Question	1	2	3	4	5	6	7	8	9	10	11	12	13	14	15	16	17	18	19	20	Σ	%
Aandahl et al., 2018 [28]	Y	Y	N	Y	Y	Y	-	Y	Y	Y	Y	Y	-	-	Y	Y	Y	N	N	Y	15	88
Aragones et al., 2018 [83]	Y	Y	N	Y	Y	Y	-	Y	Y	Y	Y	Y	-	-	N	Y	Y	N	N	Y	14	82
Busko et al., 2016 [104]	Y	Y	N	Y	Y	Y	-	Y	N	Y	Y	Y	-	-	N	Y	Y	N	N	Y	13	76
Busko and Nikolaidis, 2018 [103]	Y	Y	N	Y	Y	Y	-	Y	Y	Y	Y	Y	-	-	N	Y	Y	N	N	Y	14	82
Branco et al., 2019 [58]	Y	Y	N	Y	Y	Y	-	Y	Y	Y	Y	Y	-	-	N	Y	Y	N	N	Y	14	82
Chang et al., 2021 [59]	Y	Y	N	Y	Y	Y	-	Y	Y	Y	Y	Y	-	-	N	Y	Y	Y	N	Y	15	88
Cheng et al., 2015 [97]	Y	Y	N	Y	Y	Y	-	Y	Y	Y	Y	Y	-	-	N	Y	Y	N	N	Y	14	82
Daniel and Razvan-Liviu, 2014 [60]	Y	Y	N	Y	Y	Y	-	Y	N	Y	N	Y	-	-	N	Y	Y	N	Y	N	10	59
Di Bacco et al., 2020 [84]	Y	Y	N	Y	Y	Y	-	Y	Y	Y	Y	Y	-	-	N	Y	Y	Y	N	Y	15	88
Diniz et al., 2021 [26]	Y	Y	Y	Y	Y	Y	-	Y	Y	Y	Y	Y	-	-	N	Y	Y	Y	N	Y	16	94
Dworak et al., 1998 [27]	Y	Y	N	Y	Y	Y	-	Y	Y	N	N	Y	-	-	N	Y	Y	N	Y	N	10	59
Estevan et al., 2011 [51]	Y	Y	N	Y	Y	Y	-	Y	Y	Y	Y	Y	-	-	Y	Y	Y	N	N	Y	15	88
Estevan et al., 2012 [48]	Y	Y	N	Y	Y	Y	-	Y	Y	Y	Y	Y	-	-	Y	Y	Y	Y	N	Y	16	94
Estevan et al., 2013 [50]	Y	Y	N	Y	Y	Y	-	Y	Y	Y	Y	Y	-	-	Y	Y	Y	N	N	Y	15	88
Estevan and Falco, 2013 [44]	Y	Y	Y	Y	Y	Y	-	Y	Y	Y	Y	Y	-	-	Y	Y	Y	Y	N	Y	16	94
Estevan et al., 2014 [56]	Y	Y	N	Y	Y	Y	-	Y	Y	Y	Y	Y	-	-	Y	Y	Y	Y	N	Y	15	88
Estevan et al., 2015 [43]	Y	Y	Y	Y	Y	Y	-	Y	Y	Y	Y	Y	-	-	Y	Y	Y	Y	N	Y	17	100
Falco et al., 2009 [49]	Y	Y	N	Y	Y	Y	-	Y	N	Y	Y	Y	-	-	Y	Y	Y	Y	N	Y	15	88
Falco et al., 2013 [52]	Y	Y	N	Y	Y	Y	-	Y	Y	Y	Y	Y	-	-	Y	Y	Y	Y	N	Y	16	94
Fife et al., 2013 [75]	Y	Y	N	Y	Y	Y	-	Y	N	Y	Y	Y	-	-	N	Y	Y	Y	N	Y	14	82
Fife et al., 2013a [76]	Y	Y	N	Y	Y	Y	-	Y	Y	Y	Y	Y	-	-	N	Y	Y	Y	N	Y	15	88
Gavagan and Sayers, 2017 [24]	Y	Y	N	Y	Y	Y	-	Y	Y	Y	Y	Y	-	-	N	Y	Y	Y	N	Y	15	88
Goethel et al., 2019 [45]	Y	Y	Y	Y	Y	Y	-	Y	Y	Y	Y	Y	-	-	N	Y	Y	N	N	N	14	82
Gorski et al., 2014 [113]	Y	Y	N	Y	Y	Y	-	Y	Y	N	N	Y	-	-	N	Y	Y	N	N	N	11	65
Gorski and Orysiak, 2019 [105]	Y	Y	N	Y	Y	Y	-	Y	Y	Y	Y	Y	-	-	N	Y	Y	N	N	Y	14	82
Goulart et al., 2017 [32]	Y	Y	N	Y	Y	Y	-	Y	N	Y	Y	Y	-	-	N	Y	Y	N	N	Y	13	76
Grymanowski et al., 2019 [85]	Y	Y	N	Y	Y	Y	-	Y	Y	N	N	Y	-	-	N	N	Y	N	Y	Y	10	59
Guo, 2013 [23]	Y	y	N	Y	Y	Y	-	Y	Y	N	N	Y	-	-	N	Y	Y	N	Y	N	10	59
Huang et al., 2022 [61]	Y	Y	N	Y	Y	Y	-	Y	Y	Y	Y	Y	-	-	N	Y	Y	Y	N	Y	15	88
Ibrahim et al., 2022 [86]	Y	Y	N	Y	Y	Y	-	Y	Y	Y	Y	Y	-	-	N	Y	Y	N	N	Y	14	82
Jakubiak and Saunders, 2008 [29]	Y	Y	N	Y	Y	Y	-	Y	N	Y	Y	Y	-	-	N	Y	Y	N	Y	Y	12	71
Jandacka et al., 2013 [53]	Y	Y	N	Y	Y	Y	-	Y	Y	Y	Y	Y	-	-	Y	Y	Y	Y	N	Y	16	94
Jovanovic et al., 2020 [87]	N	Y	N	Y	Y	Y	-	Y	Y	N	N	Y	-	-	N	Y	Y	N	Y	N	9	53
Jung and Park, 2018 [63]	Y	Y	N	Y	Y	Y	-	Y	Y	Y	Y	Y	-	-	N	Y	Y	Y	Y	Y	15	88
Jung and Park, 2020 [62]	Y	Y	N	Y	Y	Y	-	Y	Y	Y	Y	Y	-	-	N	Y	Y	Y	Y	Y	14	82
Jung and Park, 2022 [46]	Y	Y	Y	Y	Y	Y	-	Y	Y	Y	Y	Y	-	-	N	Y	Y	Y	N	Y	16	94

Kim et al., 2011 [47]	Y	Y	Y	Y	Y	Y	-	Y	Y	Y	Y	Y	-	-	N	Y	Y	Y	N	Y	16	94
Kim and Kim., 2014 [64]	Y	Y	N	Y	Y	Y	-	-	Y	Y	Y	Y	-	-	N	Y	Y	Y	Y	Y	14	82
Kuragano and Yokokura, 2020 [88]	Y	Y	N	Y	Y	Y	-	Y	Y	N	N	N	-	-	N	N	Y	N	Y	N	8	47
Lee et al., 2008 [81]	N	Y	N	N	N	N	-	Y	N	N	N	Y	-	-	N	N	Y	N	Y	N	5	29
Lin et al., 2023 [25]	Y	Y	Y	Y	Y	Y	-	Y	Y	Y	Y	Y	-	-	N	Y	Y	N	N	Y	15	88
Liu et al., 2021 [2]	Y	Y	N	Y	Y	Y	-	Y	Y	Y	Y	Y	-	-	N	Y	Y	Y	N	Y	15	88
Margaritopoulos et al., 2015 [54]	Y	Y	N	Y	Y	Y	-	Y	Y	Y	Y	Y	-	-	Y	Y	N	N	Y	Y	14	82
Moreira et al., 2015 [31]	Y	Y	Y	Y	Y	Y	-	Y	Y	Y	Y	Y	-	-	Y	Y	Y	N	N	Y	16	94
Moreira et al., 2016 [12]	Y	Y	Y	Y	Y	Y	-	Y	Y	Y	Y	Y	-	-	N	Y	Y	Y	N	N	16	94
Moreira et al., 2018 [42]	Y	Y	Y	Y	Y	Y	-	Y	Y	Y	Y	Y	-	-	N	Y	Y	Y	N	Y	17	100
Moreira et al., 2021 [21]	Y	Y	Y	Y	Y	Y	-	Y	Y	Y	Y	Y	-	-	N	Y	Y	Y	N	Y	16	94
Nadzalan et al., 2021 [98]	Y	Y	N	Y	Y	Y	-	Y	Y	Y	Y	Y	-	-	N	Y	Y	N	N	Y	14	82
Nadzalan et al., 2022 [89]	Y	Y	N	Y	Y	Y	-	Y	Y	Y	Y	Y	-	-	N	Y	Y	N	N	Y	14	82
Ng and Jumadi, 2022 [108]	Y	Y	N	Y	Y	Y	-	Y	N	N	N	Y	-	-	N	Y	Y	Y	N	Y	12	71
Olsen and Hopkins, 2003 [30]	Y	Y	N	Y	N	Y		Y	Y	Y	N	Y			N	Y	Y	N	N	Y	12	71
Ortenburger et al., 2016 [90]	Y	Y	N	Y	Y	Y	-	Y	Y	Y	Y	Y	-	-	N	Y	Y	N	N	Y	14	82
Osman et al., 2022 [99]	Y	Y	N	Y	Y	Y	-	Y	Y	N	N	Y	-	-	N	Y	Y	N	N	Y	10	59
O'Sullivan et al., 2008 [67]	Y	Y	N	Y	Y	Y	-	Y	Y	Y	N	Y	-	-	N	Y	Y	N	Y	Y	12	71
O'Sullivan et al., 2009 [66]	Y	Y	N	Y	Y	Y	-	Y	Y	Y	N	Y	-	-	N	Y	Y	N	N	N	12	71
O'Sullivan and Fife, 2015 [65]	Y	Y	N	Y	Y	Y	-	Y	Y	N	Y	Y	-	-	N	Y	Y	Y	N	Y	14	82
Pedzich et al., 2006 [112]	Y	Y	N	Y	Y	Y	-	Y	Y	Y	Y	Y	-	-	N	Y	Y	N	Y	N	12	71
Pieter and Pieter, 1995 [72]	Y	Y	N	Y	Y	Y	-	Y	N	Y	Y	Y	-	-	N	Y	Y	N	Y	N	11	65
Pozo et al., 2011 [109]	Y	Y	N	Y	Y	Y	-	Y	Y	Y	Y	Y	-	-	N	Y	Y	Y	N	Y	15	88
Preuschl et al., 2016 [100]	Y	Y	N	Y	Y	Y	-	Y	Y	Y	Y	Y	-	-	N	Y	Y	Y	N	Y	15	88
Ramakrishnan et al., 2017 [110]	Y	Y	N	Y	Y	Y	-	Y	N	Y	Y	Y	-	-	N	Y	Y	N	N	Y	13	76
Rexhepi et al., 2018 [80]	Y	Y	N	Y	Y	Y	-	Y	Y	N	N	Y	-	-	N	Y	Y	N	Y	N	11	65
Serina and Lieu, 1991 [71]	Y	Y	N	Y	Y	Y	-	Y	Y	N	Y	Y	-	-	N	Y	Y	N	N	N	12	71
Sorensen et al., 1996 [91]	Y	Y	N	Y	Y	Y	-	Y	Y	N	Y	Y	-	-	N	Y	Y	Y	N	Y	14	82
Straiotto et al., 2021 [68]	Y	Y	N	Y	Y	Y	-	Y	Y	Y	Y	Y	-	-	N	Y	Y	N	N	Y	15	88
Thibordee and Prasartwuth, 2014 [22]	Y	Y	N	Y	Y	Y	-	Y	Y	Y	Y	Y	-	-	N	Y	Y	N	N	Y	14	82
Thibordee and Prasartwuth, 2014a [106]	Y	Y	N	Y	Y	Y	-	Y	Y	Y	Y	Y	-	-	N	Y	Y	Y	N	Y	15	88
Vagner et al., 2018 [111]	Y	Y	N	Y	Y	Y	-	Y	Y	Y	Y	Y	-	-	N	Y	Y	Y	N	Y	15	88
Vagner et al., 2019 [11]	Y	Y	N	Y	Y	Y	-	Y	Y	Y	Y	Y	-	-	Y	Y	Y	Y	N	Y	16	94
Vagner et al., 2022 [55]	Y	Y	N	Y	Y	Y	-	-	Y	Y	Y	Y	-	-	Y	Y	Y	Y	N	Y	16	94
Vencesbrito et al., 2014 [92]	Y	Y	N	Y	Y	Y	-	Y	Y	Y	Y	Y	-	-	N	Y	Y	N	N	Y	13	76
Wasik, 2010 [77]	Y	Y	N	Y	Y	Y	-	Y	Y	N	Y	Y	-	-	N	Y	Y	N	N	N	12	71
Wasik, 2011 [13]	Y	Y	N	Y	Y	Y	-	Y	Y	Y	Y	Y	-	-	N	Y	Y	N	N	N	13	76

Wasik, 2011a [82]	Y	Y	N	Y	Y	Y	-	Y	Y	Y	Y	Y	-	-	N	Y	Y	N	N	N	13	76
Wasik and Shan, 2015 [69]	Y	Y	N	Y	Y	Y	-	Y	Y	Y	Y	Y	-	-	N	Y	Y	N	N	Y	14	82
Wasik and Shan, 2015a [70]	Y	Y	N	Y	Y	Y	-	Y	Y	Y	Y	Y	-	-	N	Y	Y	N	N	Y	14	82
Wasik and Gora, 2016 [96]	Y	Y	N	Y	Y	Y	-	Y	Y	Y	N	Y	-	-	N	Y	Y	N	N	Y	13	76
Wasik and Gora, 2016a [93]	Y	Y	N	Y	Y	Y	-	Y	Y	Y	Y	Y	-	-	N	Y	Y	N	N	Y	14	82
Wasik et al., 2018 [95]	Y	Y	N	Y	Y	Y	-	Y	Y	Y	Y	Y	-	-	N	Y	Y	N	Y	Y	13	76
Wasik et al., 2019 [94]	Y	Y	N	Y	Y	Y	-	Y	Y	Y	Y	Y	-	-	N	Y	Y	N	N	Y	14	82
Wasik et al., 2021 [79]	Y	Y	N	Y	Y	Y	-	Y	Y	Y	Y	Y	-	-	N	Y	Y	N	Y	Y	13	76
Wasik et al., 2021a [78]	Y	Y	N	Y	Y	Y	-	Y	Y	Y	Y	Y	-	-	N	Y	Y	N	N	Y	14	82
Wasik et al., 2022 [73]	Y	Y	N	Y	Y	Y	-	Y	Y	Y	Y	Y	-	-	N	Y	Y	Y	N	Y	15	88
Wasik et al., 2023 [74]	Y	Y	N	Y	Y	Y	-	Y	Y	Y	Y	Y	-	-	N	Y	Y	N	N	Y	14	82
Wasik et al., 2023a [107]	Y	Y	N	Y	Y	Y	-	Y	Y	Y	Y	Y	-	-	N	Y	N	Y	Y	Y	13	76
Wilk et al., 1983 [57]	N	Y	N	N	N	N	-	Y	N	N	N	Y	-	-	N	Y	Y	N	N	N	6	35
Woo et al., 2013 [101]	Y	Y	N	Y	Y	Y	-	Y	Y	Y	Y	Y	-	-	N	Y	Y	Y	N	Y	15	88
Yu et al., 2012 [102]	Y	Y	N	Y	Y	Y	-	Y	Y	Y	Y	Y	-	-	N	Y	Y	N	N	N	13	76

Key: "Y" = Yes, "N" = No, "-" = Item not scored

Table S3. Summary of studies that reported kicking strike velocity

Author, year of publication	*Participants (n) (nMale, nFemale)*<br>*Age (years)*<br>*Experience (years)*<br>*Combat sport discipline*	*Measurement method*	*Strike measured*	*Reported value (Mean ± SD)*
Aandahl et al., 2018 [28]	16 (11m, 5f)<br>20.6 ± 5.5<br>3.8 ± 2.5<br>TKD, KB	Motion capture system with 6 cameras sampling at 500hz<br>Marker: Fifth metatarsus	Roundhouse kick	Vmean: 17.9 ± 2.3
Aragones et al., 2017 [83]	15 (10m, 5f)<br>41.2 ± 12.55<br>18.60 ± 7.57<br>KA	Motion capture system with 10 high speed cameras sampling at 333hz<br>Marker: Medial and lateral malleolus, first and fifth metatarsus	Front kick	Vmean: 9.20 ± 1.7
Branco et al., 2019 [58]	33 (33m)<br>Veteran: 54.2 ± 3.9, young: 23 ± 5.8<br>Veteran: 32.8 ± 10.0, young: 14.0 ± 4.6<br>KA	Motion capture system with 1 high speed cameras sampling at 210hz<br>Marker: Forefoot	Front kick	Vmean: 9.1 ± 1.7
Chang et al, 2021 [59]	12 (6m, 6f)<br>20.33 ± 1.27<br>11.25 ± 2.83<br>TKD	Motion capture system with 6 high speed cameras sampling at 100hz<br>Marker: Fifth metatarsal	Roundhouse kick<br>Roundhouse kick to head	Vmean: 10.0 ± 1.7<br>Vmean: 9.6 ± 1.7
Cheng et al., 2015 [97]	10 (10m)<br>22.00 ± 2.72<br>≥4<br>TKD	Motion capture system with 3 high speed cameras sampling at 200hz<br>Marker: Lateral malleolus	Back kick	Vmean: 10.0 ± 1.0
Daniel and Razvan-Liviu, 2014 [60]	10 (10m)<br>21-24<br>NR<br>KA	Motion capture system<br>Marker: NR	Roundhouse kick	Vmean: 12.3 ± 0.3
Di Bacco et al., 2021 [84]	16 (16f)<br>23 ± 3.7<br>0 (Untrained)<br>Krav Maga	Motion capture system with 7 high speed cameras sampling at 100hz<br>Marker: Ankle	Front kick	Vmean: 5.3 ± 0.2
Diniz et al., 2021 [26]	47 (47m)<br>25.5 ± 4.7<br>NR (“Black belt” or equivalent)<br>TKD, MT, KA	Motion capture system with 6 infra-red cameras sampling at 200hz<br>Marker: Fifth metatarsus	Roundhouse kick<br>KA<br>TKD<br>MT	 Vmean: 9.2 ± 1.2<br>Vmean: 8.0 ± 1.2<br>Vmean: 6.9 ± 1.4
Estevan et al., 2013 [50]	9 (5m, 4f)<br>26.62 ± 4.6<br>14.16 ± 5.60<br>TKD	Motion capture system with 8 cameras sampling at 247hz<br>Marker: Posterior calcaneus	Roundhouse kick	Vmean: 14.4 ± 2.7

Estevan et al., 2015 [43]	10 (5m, 5f)<br>m: 28.6 ± 2.7, f: 22.2 ±5.5<br>12.9 ±5.3<br>TKD	Motion capture system with 8 cameras sampling at 240hz<br>Marker: Posterior calcaneus	Roundhouse kick	Vmean: 11.9 ± 1.4
Fife et al., 2013 [75]	4 (2m, 2f)<br>m: 22.0 ± 0.0, f: 20.0 ± 2.8<br>NR (“expert”)<br>TKD	Motion capture system with 8 infrared cameras sampling at 500hz<br>Marker: Lateral malleolus and fifth metatarsus	Roundhouse kick to head<br>Axe kick<br>Back kick	Vmean: 16.9 ± 4.7<br>Vmean: 8.5 ± 1.9<br>Vmean: 9.9 ± 1.5
Fife et al., 2013a [76]	12 (12m)<br>22.5 ± 3.5<br>NR (“A class international”)<br>TKD	Motion capture system with 8 infrared cameras sampling at 500hz<br>Marker: Foot	Roundhouse kick to head<br>Axe kick<br>Back kick	Vmean: 11.9 ± 1.8<br>Vmean: 8.9 ± 1.7<br>Vmean: 10.6 ± 1.4
Gavagan and Sayers, 2017 [24]	24 (m/f NR)<br>TKD: 28.6 ± 9.5, MT: 22.3 ± 4.1, KA: 30.3 ± 10.7<br>≥5<br>TKD, MT, KA	Motion capture system with 7 infrared cameras sampling at 500hz<br>Marker: Lateral malleolus and fifth metatarsus	Roundhouse kick to head	Vmean: 14.7 ± 1.2
Goethel et al., 2019 [45]	14 (14m)<br>Elite: 26.3 ± 6.9, Sub-elite: 27.5 ± 6.1<br>NR (“National” to “state” level)<br>KA	Motion capture system with 7 infrared cameras sampling at 250hz<br>Marker: Lateral malleolus and fifth metatarsus	Front kick	Vmean: 9.5 ± 0.8
Goulart et al., 2016 [32]	31 (18m, 13f)<br>20.17 ± 1.89<br>NR<br>TKD	Contact mat and contact sensor in TKD Racket<br>Marker: NA	Roundhouse kick	Vmean: 9.0 ± 0.9
Grymanowski et al., 2019 [85]	1 (1m)<br>32<br>NR (“Expert”)<br>MT	Motion capture system with 6 cameras<br>Marker: Lateral malleolus and fifth metatarsus	Front kick	Vmax: 8.3*
Guo, 2013 [23]	8 (8m)<br>22.6 ± 2.9<br>NR (“Domestic master” and “first grade”)<br>TKD	Motion capture system with 2 cameras sampling at 120hz<br>Marker: NR	Back kick	Vmean: 8.9 ± 0.3
Huang et al., 2022 [61]	18 (m/f NR)<br>19.89 ± 1.02<br>10.28 ± 1.74<br>TKD	Motion capture system with 8 infrared cameras sampling at 200hz<br>Marker: Lateral malleolus and fifth metatarsus	Roundhouse kick	Vmean: 12.7 ± 1.7
Ibrahim et al., 2022 [86]	25 (25m)<br>22.57 ± 1.36<br>NR (“National level”)<br>TKD	Multiple high-speed cameras<br>Marker: Lateral malleolus and calcaneus	Front kick	Vmean: 5.2 ± 1.0

Jakubiak and Saunders, 2008 [29]	12 (12m)<br>NR<br>NR ("Experienced")<br>TKD	Floor mounted pressure sensor and sensor house in TKD racket<br>Marker: NA	Roundhouse kick	Vmean: 11.8 ± 0.7*
Jandacka et al., 2013 [53]	10 (5m, 5f)<br>25.4 ± 5.0<br>13.8 ± 5.8<br>TKD	Motion capture system with 8 cameras sampling at 247hz<br>Marker: Posterior calcaneus	Roundhouse kick	Vmean: 12.0 ± 1.2
Jovanovic et al., 2020 [87]	16 (16m)<br>18-22<br>NR<br>KA	Camera based motion capture system<br>Marker: NR	Front kick	Vmean: 16.7 ± 1.3
Jung and Park, 2018 [63]	10 (10m)<br>21.7 ± 0.5<br>8.9 ± 1.1<br>TKD	Motion capture system with 7 high speed cameras sampling at 250hz<br>Marker: Lateral malleolus, fifth metatarsus and calcaneus	Roundhouse kick	Vmean: 12.8 ± 1.1
Jung and Park, 2020 [62]	10 (10m)<br>21.7 ± 0.5<br>8.9 ± 1.1<br>TKD	Motion capture system with 7 high speed cameras sampling at 250hz<br>Marker: Lateral malleolus, fifth metatarsus and calcaneus	Roundhouse kick	Vmean: 16.8 ± 1.3
Jung and Park, 2022 [46]	10 (10m)<br>21.7 ± 0.5<br>8.9 ± 1.1<br>TKD	Motion capture system with 7 infrared cameras sampling at 250hz<br>Marker: Lateral malleolus, fifth metatarsus and calcaneus	Roundhouse kick	Vmean: 15.6 ± 2.2
Kim and Kim, 2014 [64]	12 (12m)<br>20.4 ± 8.4<br>10.6 ± 3.2<br>TKD	Motion capture system with 6 cameras sampling at 200hz<br>Marker: Lateral malleoli	Roundhouse kick<br>Back kick	Vmean: 15.9 ± 1.7<br>Vmean: 12.2 ± 0.8
Kim et al., 2011 [47]	12 (12m)<br>20.4 ± 8.4<br>10.6 ± 3.2<br>TKD	Motion capture system with 6 cameras sampling at 200hz<br>Marker: Lateral malleoli	Roundhouse kick<br>Back kick<br>Thrashing kick<br>Turning back kick	Vmean: 14.7 ± 1.3<br>Vmean: 11.5 ± 1.0<br>Vmean: 9.9 ± 1.3<br>Vmean: 11.4 ± 1.2
Kuragano and Yokokura, 2012 [88]	1 (1m)<br>26<br>NR ("2nd degree black belt")<br>Nihon-Kempo	High speed video system, 125 frames per second<br>Marker: Toes	Front kick	Vmax: 9.2*
Lee et al., 2008 [81]	1 (1m)<br>NR<br>NR ("High-level male")<br>TKD	A tripod mounted camera<br>Marker: Foot	Side kick	Vmax: 12.7

Lin et al., 2023 [25]	20 (20m)<br>19.90 ± 0.97<br>10.25 ± 1.77<br>TKD	Motion capture system with 8 cameras sampling at 200hz<br>Marker: Medial ankle, heel, toes	Side kick	Vmean: 6.3 ± 0.2
Liu et al., 2021 [2]	19 (19m)<br>19.90 ± 0.98<br>10.36 ± 1.73<br>TKD	Motion capture system with 8 cameras sampling at 200hz<br>Marker: Lateral malleolus	Roundhouse kick	Vmean: 11.6 ± 1.6
Moreira et al., 2015 [31]	6 (6m)<br>20.5 ± 4.3<br>≥6<br>TKD	Motion capture system with 9 cameras sampling at 200 hz<br>Marker: Foot	Roundhouse kick	Vmean: 16.1 ± 1.7
Moreira et al., 2016 [12]	14 (10m, 4f)<br>Elite: 23.6 ±2.1, Sub-elite: 22.4 ± 1.3<br>Elite: 12.2 ± 8.5, Sub-elite: 10.4 ± 6.1<br>TKD	Motion capture system with 7 cameras sampling at 250 hz<br>Marker: Posterior calcaneus, lateral malleolus and 2nd metatarsus	Roundhouse kick	Vmean: 16.8 ± 0.5*
Moreira et al., 2018 [42]	14 (10m, 4f)<br>Elite: 23.6 ± 2.1, Sub-elite: 22.4 ± 1.3<br>Elite: 12.2 ± 8.5, Sub-elite: 10.4 ± 6.1<br>TKD	Motion capture system with 7 cameras sampling at 250hz<br>Marker: Posterior calcaneus, lateral malleolus and 2nd metatarsus	Roundhouse kick	Vmean: 16.3 ± 1.6
Moreira et al., 2021 [21]	14 (10m, 4f)<br>Elite: 23.6 ± 2.1, Sub-elite: 22.4 ± 1.3<br>Elite:7.92, Sub-elite: 8.5<br>TKD	Motion capture system with 7 cameras sampling at 250 hz<br>Marker: Posterior calcaneus, lateral malleolus and 2nd metatarsus	Roundhouse kick	Vmean: 17.4 ± 0.7
Nadzalan et al., 2021 [98]	24 (m/f NR)<br>22.19 ± 1.34<br>NR ("State or university level")<br>TKD	Motion capture system with 6 infrared cameras sampling at 100hz<br>Markers: second metatarsal, lateral malleolus, calcaneus	Axe kick	Vmean: 8.7 ± 0.9
Nadzalan et al., 2022 [89]	24 (m/f NR)<br>22.19 ± 1.34<br>NR ("State or university level")<br>Taekwondo	Motion capture system with 6 infrared cameras sampling at 100hz<br>Markers: second metatarsal, lateral malleolus, calcaneus	Front kick	Vmean: 7.4 ± 0.2
Ortenburger et al., 2016 [90]	6 (6f)<br>19.8 ±. 3.8<br>NR<br>TKD	Motion capture system with 10 near-infrared cameras sampling at 370hz<br>Marker: NR	Front kick	Vmean: 9.0 ± 1.5
Osman et al., 2022 [99]	30 (m/f NR)<br>22.21 (mean)<br>≥3<br>TKD	Motion capture system with 6 infrared cameras sampling at 100hz<br>Markers: second metatarsal, lateral malleolus, calcaneus	Axe kick	Vmean: 6.7 ± 0.8

O'Sullivan and Fife, 2015 [65]	6 (3m, 3f)<br>22 ± 5<br>≥10<br>TKD (Paralympic)	Motion capture system with 7 infrared cameras sampling at 150hz<br>Marker: NR	Back kick<br>Side kick<br>Roundhouse kick	Vmean: 10.7 ± 0.5<br>Vmean: 8.0 ± 1.1<br>Vmean: 13.8 ± 1.5
O'Sullivan et al., 2008 [67]	5 (m/f NR)<br>NR<br>≥10<br>TKD	Motion capture system with 7 cameras sampling at 150 hz<br>Marker: NR	Roundhouse kick<br>Roundhouse kick to head	Vmean: 17.7 ± 1.7<br>Vmean: 16.5 ± 0.6
O'Sullivan et al., 2009 [66]	10 (m/f NR)<br>NR<br>≥10<br>Yongmundo and TKD	Motion capture system with 7 cameras sampling at 150 hz<br>Marker: NR	Roundhouse kick<br>Roundhouse to head	Vmean: 18.3 ± 1.1<br>Vmean: 17.2 ± 0.5
Pieter and Pieter, 1995 [72]	26 (11m, 15f)<br>m: 21.8±3.7, 23.3±5.0, f: 25.6±5.2, 26.5±5.5<br>NR ("Olympic team")<br>TKD	Electronic dual beam timing system 1cm from impact surface<br>Marker: NA	Side kick<br>Roundhouse kick<br>Back kick	Vmean: 6.9 ± 0.4<br>Vmean: 16.3 ± 1.3<br>Vmean: 9.1 ± 1.5
Preuschl et al., 2016 [100]	22 (22m)<br>23 ± 5.3<br>≥4<br>TKD	Motion capture system with 8 infrared cameras sampling at 250hz<br>Markers: second metatarsal, lateral malleolus, calcaneus	Axe kick	Vmean: 7.8 ± 0.9
Rexhepi et al., 2018 [80]	7 (7m)<br>NR<br>NR ("Karate master")<br>KA	Motion capture system with 3 cameras sampling at 60hz<br>Marker: NR	Roundhouse kick to head	Vmean: 14.0 ± 2.3
Serina and Lieu, 1991 [71]	3 (3m)<br>NR<br>NR ("Black belt (1st Dan))"<br>TKD	Motion capture system with 2 infrared cameras sampling at 500hz<br>Marker: base of the foot below the calcaneum, first metatarsal	Roundhouse kick<br>Side kick<br>Back Kick	Vmax: 15.6*<br>Vmax: 8.3*<br>Vmax: 9.4*
Sorensen et al., 1996 [91]	17 (13m, 4f)<br>18-34<br>≥3<br>TKD	Motion capture system with 1 camera sampling at 200hz<br>Marker: lateral malleolus, fifth metatarsal joint	Front kick	Vmax: 11.7*
Straiotto et al., 2021 [68]	18 (17m, 1f)<br>Elite: 27.0 ± 0.4, non-elite: 35.0 ± 0.1<br>Elite: 18.8 ± 1.5, non-elite: 8.3 ± 1.0<br>TKD	Motion capture system with 8 camera sampling at 500hz<br>Marker: fifth metatarsus and calcaneus	Roundhouse kick	Vmax: 13.6 ± 0.6

Vagner et al., 2019 [11]	25 (25m)<br>27.7 ± 7.2<br>≥2<br>Military combat	Motion capture system with 6 camera sampling at 500hz<br>Marker: lateral malleolus	Front kick	Vmean: 7.7 ± 1.03
Vagner et al., 2022 [55]	24 (24m)<br>26.8 ± 10.1<br>NR<br>Military combat	Motion capture system with 6 camera sampling at 200hz<br>Marker: lateral malleolus	Front kick	Vmean: 8.2 ± 0.92
VencesBrito et al., 2014 [92]	30 (30m)<br>KA: 24 ± 7, untrained: 23 ± 6<br>KA: 13 (mean), untrained: 0<br>KA and untrained participants	Motion capture system with 1 camera sampling at 210hz<br>Marker: Markers: second metatarsal, lateral malleolus	Front kick	Vmean: 8.1 ± 0.9
Wasik, 2010 [77]	1 (NR)<br>17<br>NR ("International Sports Master")<br>TKD	Motion capture system with 6 infrared cameras sampling at 120hz<br>Marker: NR	Roundhouse kick to head	Vmax: 10.4
Wasik, 2011 [13]	6 (5m, 1f)<br>16.5 ± 0.7<br>≥4<br>TKD	Motion capture system with 6 infrared cameras sampling at 120hz<br>Marker: NR	Side kick	Vmean: 5.7 ± 1.2
Wasik, 2011a [82]	1 (1m)<br>17<br>NR ("1st Dan")<br>TKD	Motion capture system with 6 infrared cameras sampling at 120hz<br>Marker: NR	Side kick	Vmean: 5.6 ± 0.4
Wasik and Shan, 2015 [69]	6 (5m, 1f)<br>16.5 ± 0.7<br>≥4<br>TKD	Motion capture system with 6 infrared cameras sampling at 120hz<br>Marker: NR	Roundhouse kick	Vmean: 9.8 ± 2.5<br>Vmax: 14.6
Wasik and Shan, 2015a [70]	6 (5m, 1f)<br>16.5 ± 5<br>≥4<br>TKD	Motion capture system with 6 infrared cameras sampling at 120hz<br>Marker: NR	Roundhouse kick	Vmean: 14.6 ± 0.7
Wasik and Gora, 2016 [96]	3 (m/f NR)<br>16.3 ± 0.6<br>2-6<br>TKD	Motion capture system with 6 infrared cameras sampling at 120hz<br>Marker: NR	Back kick	Vmean: 6.00 ± 1.4<br>Vmax: 7.34
Wasik and Gora, 2016a [93]	1 (1m)<br>28<br>NR ("2nd Dan Black Belt")<br>TKD	Motion capture system with 10 camera sampling at 370hz<br>Marker: NR	Front kick	Vmean: 13.9 ± 0.9

Wasik et al., 2018 [95]	14 (8m, 6f)<br>m: 18.3 ± 1.7, f: 19.8 ± 3.8<br>≥4<br>TKD	Motion capture system with 10 camera sampling at 250hz<br>Marker: metatarso- phalangeal joint	Front kick	Vmean: 11.0 ± 1.2
Wasik et al., 2019 [94]	14 (8m, 6f)<br>m: 18.3 ±1.7, f: 19.8 ± 3.8 y<br>≥4<br>TKD	Motion capture system with 10 near-infrared camera sampling at 370hz<br>Marker: NR	Front kick	Vmean: 10.8 ± 1.3
Wasik et al., 2021 [79]	15 (15m)<br>22.5 ± 6.2<br>10.67<br>TKD	Motion capture system with 10 near-infrared camera sampling at 370hz<br>Marker: lateral side of the foot	Roundhouse kick to head	Vmax: 14.6
Wasik et al., 2021a [78]	15 (15m)<br>22.5 ± 6.2<br>≥4<br>TKD	Motion capture system with 10 near-infrared camera sampling at 370hz<br>Marker: dorsal part of the foot	Roundhouse kick to head<br>Front kick	Vmean: 14.6 ± 1.5*<br>Vmean: 10.4 ± 1.2*
Wasik et al., 2022 [73]	15 (15m)<br>21.9 ± 6.8 y<br>≥4<br>TKD	Motion capture system with 10 near-infrared camera sampling at 370hz<br>Marker: Ankle	Roundhouse kick	Vmean: 12.4 ± 2.0
Wasik et al., 2023 [74]	13 (m/f NR)<br>22.6 ± 6.28<br>≥4<br>TKD	Motion capture system with 10 near-infrared camera sampling at 370hz<br>Marker: Great toe	Roundhouse kick	Vmean: 13.2 ± 2.3<br>Vmax: 16.6
Wilk et al., 1983 [57]	NR<br>NR<br>NR<br>KA	Multiflash strobe lamp at 60 or 120 flashes per second<br>Marker: NR	Roundhouse kick<br>Back kick<br>Front kick<br>Side kick	Vmean: 10.3 ± 0.8<br>Vmean: 11.3 ± 0.7<br>Vmean: 12.2 ± 3.2<br>Vmean: 12.2 ± 3.2
Woo et al., 2013 [101]	15 (m/f NR)<br>NR<br>≥10<br>TKD	Motion capture system with 9 cameras sampling at 150hz<br>Marker: fifth metatarsal	Front axe kick<br>In-out axe kick<br>Out-in axe kick	Vmean: 7.9 ± 1.1<br>Vmean: 7.7 ± 0.6<br>Vmean: 7.4 ± 0.7<br>Vmax: 11.4
Yu et al., 2012 [102]	12 (12m)<br>Professional: 23.4 ± 1.2, advanced: 21.3 ± 1.6<br>Professional: ≥15, advanced: ≥5<br>TKD	Motion capture system with 4 camera sampling at 60hz<br>Marker: Ankle	Axe kick	Vmean: 10.9 ± 1.2

*KEY: NR = not reported, TKD = Taekwondo, KA = Karate, KB = Kickboxing, MT = Muay Thai, Vmean = mean velocity reported, Vmax = maximum velocity reported, * = Extracted from figure*

Table S4. Summary of studies that reported kicking strike impact force

Author, year of publication	*Participants (n) (nMale, nFemale)*<br>*Age (years)*<br>*Experience (years)*<br>*Combat sport discipline*	*Measurement method*	*Strike measured*	*Reported value (Mean ± SD)*
Busko and Nikolaidis., 2018 [103]	6 (6m)<br>17.7 ± 0.7<br>6.5 ± 1.6<br>TKD	BTS-3 (Dynamometric punching bag with embedded strain gauge)	Roundhouse kick<br>Back kick	Fmean: 4580.8 ± 1130.3<br>Fmean: 3568.0 ± 1306.0
Busko et al., 2016 [104]	28 (14m, 14f)<br>m: 17.6 ± 2.7, f: 18.7 ± 3.1<br>m: 8.1 ± 2.8, f: 7.1 ± 4.5<br>≥7.1<br>TKD	BTS-4AP-2K (Dynamometric punching bag embedded with 2 two tri-axial accelerometers)	Roundhouse kick<br>Back kick	Fmean: 2072.3 ± 472.2<br>Fmean: 3514.6 ± 1190.4
Di Bacco et al., 2021 [84]	16<br>23 ± 3.7<br>0 (Untrained)<br>Krav Maga	Vertically mounted impact force plate with foam padding	Front kick	Fmax: 2247*
Dworak et al., 1998 [27]	26 (m/f NR)<br>KA: 29.4 ± 4.9, KB: 21.9 ± 2.1<br>KA: 12.6 ± 4.1, KB: 3.3 ± 2.1<br>KA and KB	Three axis piezoelectric force platform covered with absorbing cushion	Front kick<br>Side kick	Fmean: 68.8 ± 26.9 N/KG<br>Fmean: 91.2 ± 14.8 N/KG
Estevan and Falco, 2013 [44]	33 (33m)<br>24.3 ± 3.0<br>≥4<br>TKD	A force platform was placed with 9 piezoresistant sensors organised in a triangular structure on a striking dummy.	Roundhouse kick<br>Roundhouse kick to head	Fmean: 19.8 ± 6.6 N/KG<br>Fmean: 18.7 ± 3.5 N/KG
Estevan et al., 2011 [51]	27 (27m)<br>26.56 ± 2.23<br>≥4<br>TKD	A force platform was placed with 5 piezoresistant sensors organised in a pentagonal structure on a striking dummy.	Roundhouse kick to head	Fmean: 1829 ±161
Estevan et al., 2012 [48]	36 (36m)<br>25.03 ± 5.68<br>≥4<br>TKD	A force platform was placed with 9 piezoresistant sensors organised in a triangular structure on a striking dummy.	Roundhouse kick to head	Fmean: 1464.4 ± 448.9
Estevan et al., 2014 [56]	43 (33m, 10f)<br>24.4 ± 5.4<br>10.8 9 ± 6.88<br>TKD	A force platform was placed with 9 piezoresistant sensors organised in a triangular structure on a striking dummy.	Roundhouse kick	Fmean: 1277 ± 504.1

Falco et al., 2009 [49]	31 (m/f NR)<br>21.57 ± 4.75<br>≥4<br>TKD	A force platform was placed with 5 piezoresistant sensors organised in a pentagonal structure on a striking dummy.	Roundhouse kick	Fmean: 2089.8 ± 634.7
Falco et al., 2013 [52]	49 (34m, 14f)<br>m expert: 23.5 ± 3.3, f expert: 23.9 ± 6.2, m novice: 25.2 ± 7.1, f novice: 25.0 ± 6.5<br>≥4<br>TKD	A force platform was placed with 5 piezoresistant sensors organised in a pentagonal structure on a striking dummy.	Roundhouse kick	Fmean: 19.3 ± 6.7 N/KG
Gavagan and Sayers, 2017 [24]	24 (m/f NR)<br>TKD: 28.6 ± 9.5, MT: 22.3 ± 4.1, KA: 30.3 ± 10.7<br>≥5<br>TKD, MT, KA	Kicking rig with embedded strain gauge, recording at 1000hz	Roundhouse kick to head<br>TKD<br>MT<br>KA	 Fmean: 1547 ± 530<br>Fmean: 1400 ± 419<br>Fmean: 1211 ± 219
Gorski et al., 2014 [113]	1 (1m)<br>25<br>NR ("2nd degree black belt")<br>TKD	BTS-4AP-2K (Dynamometric punching bag embedded with 2 two tri-axial accelerometers)	Axe kick	Fmean: 122.6 ± 14.5
Gorski and Orysiak, 2019 [105]	6 (m/f NR)<br>20.0 ± 3.2<br>NR ("Olympic taekwondo team")<br>TKD	BTS-4AP-2K (Dynamometric punching bag embedded with 2 two tri-axial accelerometers)	Roundhouse kick<br>Side kick	Fmean: 2733 ± 748<br>Fmean: 1779 ± 372
Kuragano and Yokokura, 2012 [88]	1 (1m)<br>26<br>NR ("2nd degree black belt")<br>Nihon-Kempo	A piezoelectric three-component force sensor	Front kick	Fmax: 4500
Lee et al., 2008 [81]	1 (1m)<br>NR<br>NR ("High-level male")<br>TKD	Spring balance	Side kick	Fmax: 1280
Margaritopoulos et al., 2015 [54]	10 (5m, 5f)<br>m: 18.4 ± 1.2, f: 19.2 ± 0.4<br>5 ± 0.5<br>KA	Vertically mounted force plate	Back kick	Fmean: 562.4 ± 49.5
Ng and Jumadi, 2022 [108]	4 (4m)<br>NR<br>NR<br>Silat	Force sensor attached to the surface of a kicking pad	Front kick	Fmean: 466.6 ± 19.8
Olsen and Hopkins, 2003 [30]	30 (18m, 4f, 8NR)<br>Experimental: m 26 ± 6, f 27 ±4, Control: m 27 ±11, f 27 ± 2<br>≥1<br>NR ("Martial arts")	Vertically mounted impact force plate	Front kick<br>Side kick	Fmean: 20 ± 5 N/KG<br>Fmean: 20 ± 7 N/KG

O’Sullivan et al., 2008 [67]	5 (m/f NR)<br>NR<br>≥10<br>TKD	Sandbag embedded with two tri-axial accelerometers	Roundhouse kick<br>Roundhouse kick to head	Fmean: 6400 ± 898<br>Fmean: 5419 ± 659
O’Sullivan et al., 2009 [66]	10 (m/f NR)<br>NR<br>≥10<br>Yongmundo and TKD	Sandbag embedded with two tri-axial accelerometers	Roundhouse kick<br>Roundhouse kick to head	Fmean: 6400 ± 898<br>Fmean: 5475 ± 1293
Pedzich et al., 2006 [112]	5 (m/f NR)<br>25 ± 3.74<br>5-7<br>TKD	Vertically mounted impact force plate with absorption layer	Side kick<br>Back kick	Fmean: 9015 ± 2382<br>Fmean: 8569±2381
Pieter and Pieter, 1995 [72]	26 (11m, 15f)<br>m: 21.8±3.7, 23.3±5.0, f: 25.6±5.2, 26.5±5.5<br>NR (“Olympic team”)<br>TKD	Water filled heavy bag with built in force sensor	Side kick<br>Roundhouse kick<br>Back kick	Fmean: 461.8 ± 100.7<br>Fmean: 518.7 ± 96.3<br>Fmean: 661.9 ± 52.7
Pozo et al., 2011 [109]	17 (m/f NR)<br>International: 24.2 ± 10.6, National: 37.9 ± 9.0<br>International: 13.0 ± 6.4, National: 18.7 ± 12.1<br>KA	Vertically mounted Strain gauge force plate	Front kick	Fmean: 57.7 ± 30.3 N/KG
Ramakrishnan et al., 2017 [110]	52 (42m, 10f)<br>Trained: 31.6 ± 10.3, Untrained: 28.1 ± 7.2<br>NR<br>Various martial arts and untrained participants	Vertically mounted force plate covered with high density foam	Front kick	Fmean: 5200<br>Fmax: 7790
Thibordee and Prasartwuth, 2014 [22]	20 (20m)<br>NR<br>NR (“National level”)<br>TKD	A square-shaped kicking target with polyurethane foam attached to a force transducer	Roundhouse kick	Fmean: 172.0 ± 19.4
Thibordee and Prasartwuth, 2014a [106]	16 (16m)<br>High impact: 24.3 ± 5.9, Low impact: 16.8 ± 7.7<br>High impact: 11.0 ± 7.6, Low impact: 6.5 ± 3.0<br>TKD	A rectangular-shaped kicking target with spongy foam and polyvinyl chloride sheet cover was fixed to a wall and attached to a mono- axial force transducer	Roundhouse kick	Fmean: 1490.8 ± 274.5<br>Fmax: 1986
Vagner et al., 2018 [111]	6 (6m)<br>22.2 ± 1.5<br>NR<br>Military combat	Vertically mounted force plate covered with a kick pad	Front kick	Fmean: 3180 ± 647
Vagner et al., 2019 [11]	25 (25m)<br>27.7 ± 7.2<br>≥2	A vertically anchored force plate	Front kick	Fmean: 2202 ± 489

	Military combat			
Vagner et al., 2022 [55]	24 (24m)<br>26.8 ± 10.1<br>NR<br>Military combat	Vertically mounted force plate	Front kick	Fmean: 3013 ± 824
Wasik, 2011 [13]	6 (5m, 1f)<br>16.5 ± 0.7<br>≥4<br>TKD	Calculation from motion capture	Side kick	Fmax: 1020
Wasik, 2011a [82]	1 (1m)<br>17<br>NR (“1st Dan”)<br>TKD	Calculation from motion capture	Side kick	Fmax: 1120
Wasik et al., 2023a [107]	1 (1m)<br>32<br>NR (“Master”)<br>TKD	Martial arts shield mounted on a force plate	Front kick<br>Side kick<br>Roundhouse kick	Fmean: 2008.6 ± 284.8<br>Fmean: 2406.9 ± 299.8<br>Fmean: 2330.7 ± 71.2

*KEY: NR = not reported, TKD = Taekwondo, KA = Karate, KB = Kickboxing, MT = Muay Thai, Fmean = mean impact force reported, Fmax = maximum impact force reported, * = Extracted from figure*
